# A practical guide for nephrologist peer reviewers: understanding and appraising Mendelian randomization studies

**DOI:** 10.1080/0886022X.2024.2445763

**Published:** 2025-01-13

**Authors:** Jianbo Qing, Yafeng Li, Karim M. Soliman, Wisit Cheungpasitporn

**Affiliations:** a Department of Nephrology, Sir Run Run Shaw Hospital, Zhejiang University School of Medicine, Hangzhou, China; b Department of Nephrology, Shanxi Provincial People’s Hospital (Fifth Hospital), Shanxi Medical University, Taiyuan, China; c Department of Medicine, Division of Nephrology, Medical University of South Carolina, Charleston, South Carolina, USA; d Medical Services, Ralph H. Johnson VA Medical Center, Charleston, South Carolina, USA; e Division of Nephrology and Hypertension, Department of Medicine, Mayo Clinic, Rochester, Minnesota, USA

**Keywords:** Mendelian Randomization, nephrology, kidney diseases, genetic epidemiology, causal inference, peer review

## Abstract

Identifying risk factors for disease onset and progression has been a core focus in nephrology research. Mendelian Randomization (MR) has emerged as a powerful genetic epidemiological approach, utilizing genome-wide association studies (GWAS) to establish causal relationships between modifiable risk factors and kidney disease outcomes. MR uses genetic variants as instrumental variables to infer causal relationships between exposures and disease outcomes. This method leverages the natural randomization of genetic variants to balance confounders, akin to matched cohorts in observational research. The rapid increase in MR studies on kidney disease poses challenges for journals and peer reviewers, especially clinicians unfamiliar with the methodology. High-quality MR studies use strong, well-validated genetic instruments with clear biological relevance, thoroughly testing for pleiotropy and confounding factors using methods like MR-Egger. Sensitivity analyses, such as MR-PRESSO, should ensure findings remain consistent across various assumptions. Effect sizes with confidence intervals should be reported and discussed within established biological mechanisms. Additionally, limitations must be transparently addressed, with recommendations for replication in future studies, to strengthen findings. This article guides readers in understanding MR application in nephrology and identifying high-quality MR studies, helping peers avoid pitfalls while seizing new opportunities in advancing kidney disease research.

## 1. Introduction

The prevalence of kidney diseases is steadily rising worldwide, with limited therapeutic options available for most conditions. This limitation largely stems from our incomplete understanding of disease pathogenesis and progression factors [1–4]. Mendelian Randomization (MR) has emerged as a powerful approach to address this knowledge gap, using genetic variants as instrumental variables to infer causal relationships between exposures and outcomes while mitigating confounding effects. However, the surge in MR studies on nephrology presents new challenges for peer reviewers, particularly clinicians less familiar with genetic epidemiology methodologies [3,5–23]. This educational article is designed to address these challenges by equipping nephrologists and peer reviewers with practical tools to critically evaluate MR studies, thereby enhancing their ability to discern methodological rigor and clinical relevance.

The introduction of the STROBE-MR checklist aimed to address this issue by providing a structured approach to documenting study design, methodology, and interpretation of findings [24]. However, as the checklist relies on information subjectively provided by researchers, it does not guarantee that readers can make accurate assessments. While the checklist improves transparency, it falls short in aiding the identification of potential biases and limitations within studies [3,25].

This editorial provides nephrologists, researchers, and peer reviewers with a comprehensive yet practical guide to understanding and appraising MR studies. By offering structured guidance, we aim to bridge the gap between foundational knowledge and advanced critical appraisal skills, supporting the continued advancement of high-quality research in nephrology.

## 2. The need for peer review in MR studies

**Figure 1.**
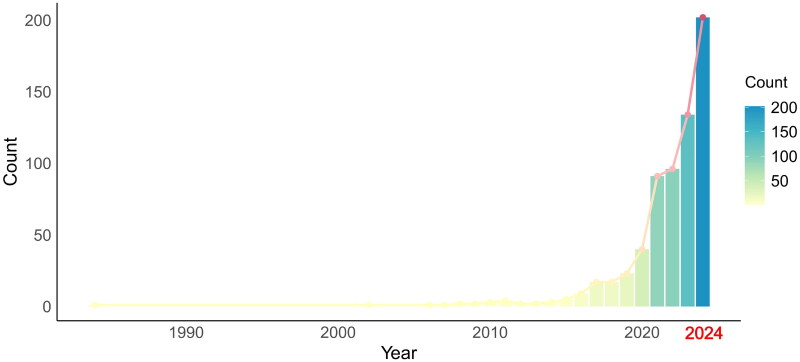
Number of publications identified using the keywords ‘Mendelian Randomization and kidney’. Data were obtained from PubMed (https://pubmed.ncbi.nlm.nih.gov) using the keywords ‘Mendelian Randomization and kidney,’ with the search cutoff date being October 13, 2024.

The rapid increase in MR studies, especially over the past three years, has significantly impacted the peer review process in nephrology (Figure 1). This growth has created challenges for peer reviewers, many of whom are clinicians with limited exposure to genetic epidemiology. The quality of current MR studies varies considerably, highlighting the need for careful evaluation to maintain scientific rigor.

Overall, the quality of current MR studies in nephrology is highly variable. Due to the straightforward nature of the methodology, MR studies are often conducted with minimal training, resulting in an influx of low-quality submissions to editorial offices. At times, studies lacking substantive contributions may even pass the peer review process. Nevertheless, MR research has revitalized the field of nephrology, and it is now up to peer reviewers to carefully discern and select high-quality studies that can truly advance nephrology research.

MR studies can be broadly categorized into exploratory and confirmatory research. Exploratory studies typically examine the relationship between a broad category of factors—such as gut microbiota, metabolites, or inflammatory markers—and a disease [18,26]. In contrast, confirmatory studies usually follow a series of investigations in which researchers have already identified one or several specific factors, such as genes or proteins, that are associated with a disease, and use MR to further clarify and enhance the precision of these associations [27,28]. As with any type of research, the topic of an MR study must be grounded in rigorous scientific inquiry and clinical relevance. Studies without practical clinical relevance, such as examining a hypothetical ‘causal link between noodle consumption and acute kidney injury,’ should not proceed to peer review. Due to the potential for residual confounding in MR studies, even absurd hypotheses can sometimes yield seemingly meaningful results. Additionally, the interventional potential of a hypothesis is a key factor in determining its clinical relevance. Studies focusing on factors that can be effectively targeted in clinical treatment or prevention, such as blood pressure or gut microbiota, should be prioritized [29].

This guide is designed to help bridge the knowledge gap for peer reviewers by offering practical insights into evaluating MR studies. By following a structured approach, peer reviewers can identify strengths and weaknesses in study design, ensuring that only high-quality research contributes to the field’s body of evidence.

## 3. Core assumptions of Mendelian randomization

MR uses the natural random allocation of genetic variants from parents to offspring to mimic the randomization process seen in clinical trials. This inherent “gating” of genetic inheritance results in individuals being naturally grouped by their genetic predispositions, enabling researchers to infer causal relationships between exposures and outcomes while minimizing confounding and reverse causation. By using genetic variants as instrumental variables, MR creates a framework in which these genetic tools are strongly associated with the exposure of interest but remain unrelated to confounding variables, thereby facilitating causal inference [30].

Instrumental variables (IVs) are central to MR and play a crucial role in establishing causality between an exposure and an outcome. For an IV to be valid, it must satisfy three core assumptions: it must have a strong association with the exposure, be independent of any confounders, and influence the outcome solely through the exposure pathway (without direct effects or pleiotropy) [31,32].

A key component of assessing MR studies is understanding the core assumptions that validate the method: relevance, independence, and the exclusion restriction. These assumptions are foundational because they directly affect the reliability of the study’s conclusions.

- **Relevance of Genetic Instruments:** This assumption states that the genetic instruments used (e.g., SNPs) must be strongly associated with the exposure of interest. For example, SNPs related to serum uric acid levels must have proven associations from large-scale GWAS. When the relevance assumption is violated, the instruments may be too weak to infer causality, leading to biased or null findings [33].
- **Independence from Confounders:** This assumption requires that the genetic instruments must not be associated with confounding factors that could bias the exposure-outcome relationship. For instance, an SNP chosen for studying serum uric acid and CKD progression should not be linked to variables like age or smoking status that may independently influence both the exposure and outcome. Failing this assumption compromises the study’s ability to draw valid causal inferences [34].
- **Exclusion Restriction and Pleiotropy:** This is perhaps the most challenging assumption to fulfill and implies that the genetic instrument affects the outcome only through the exposure and not via alternative pathways (i.e., no pleiotropy). For example, an SNP related to serum uric acid should not influence CKD progression through an unrelated biological mechanism. Statistical methods like MR-Egger and MR-PRESSO address horizontal pleiotropy, ensuring robust causal inference [35–37].

**Figure 2.**
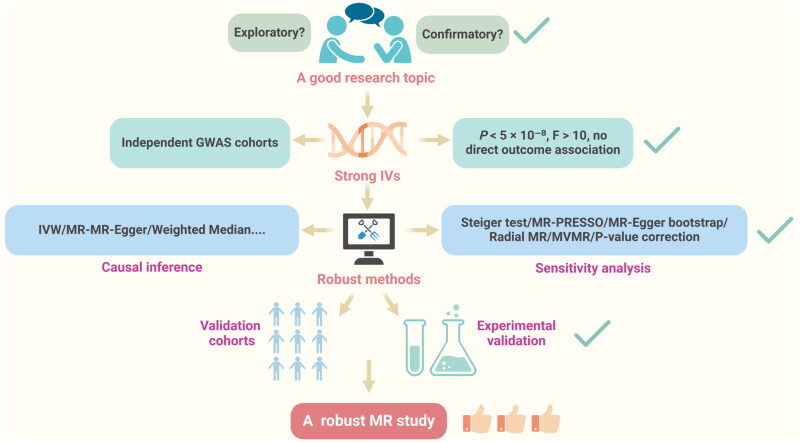
Schematic representation of high-quality MR studies. Essential components of high-quality MR studies include selecting a relevant and well-defined research topic, identifying strong IVs based on rigorous criteria, and applying robust methodologies for causal inference. Additionally, these studies incorporate thorough sensitivity analyses and validation steps, ensuring reliable and reproducible findings.

Once clinical relevance is established (Figure 2), attention must turn to cohort selection and IVs as these fundamentally determine study validity [38]. The study population should maintain ancestral homogeneity, with strictly non-overlapping exposure and outcome cohorts to ensure statistical independence [39,40]. The cohort should consist of individuals from the same ancestry, and the exposure and outcome cohorts must be distinct [41]. For example:

1. In an MR study on the causal relationship between serum uric acid levels and CKD progression, the exposure cohort should be from GWAS on serum uric acid, while the outcome cohort should be a distinct group of CKD patients [42].
2. Similarly, in an MR study examining the potential causal effect of vitamin D levels (exposure) on the incidence of acute kidney injury (AKI) (outcome), the exposure cohort could be drawn from a GWAS on vitamin D levels, while the outcome cohort would be a distinct group of patients with data on AKI occurrence.

In both cases, it’s crucial that the exposure and outcome cohorts do not overlap to avoid bias in the results.

Clarifying these assumptions is crucial as each affects the study’s conclusions. Violation of the exclusion restriction can bias causal estimates due to pleiotropy, leading to incorrect conclusions [43]. Understanding how these assumptions interact and impact results allows reviewers and researchers to critically assess the robustness of MR studies. Structured sensitivity analyses, such as those employing MR-Egger and MR-PRESSO, can provide insight into the validity of these assumptions and help interpret findings more accurately.

## 4. Selecting robust genetic instrumental variables

The selection of genetic IVs is a critical determinant of the validity and strength of MR studies. IVs, typically single nucleotide polymorphisms (SNPs), must meet three fundamental criteria to be considered robust: they must be strongly associated with the exposure of interest, must not be associated with confounding factors, and must influence the outcome solely through the exposure [44]. For instance, in an MR study investigating the causal effect of elevated serum uric acid levels on chronic kidney disease (CKD) progression, SNPs related to genes such as *SLC2A9* or *ABC transporter genes* could be used as IVs due to their established association with uric acid metabolism (*p* < 5 × 10^−8^) [45,46].

Scientific evidence supporting the strength and validity of chosen IVs can be derived from large-scale genome-wide association studies (GWAS). For example, a GWAS study identified strong associations between certain SNPs and serum urate levels, demonstrating their potential as IVs in MR research [47]. The robustness of these IVs should be reinforced through metrics such as F-statistics, with values exceeding 10 being considered sufficient to avoid weak instrument bias. In the context of nephrology, where traits like serum urate and genetic predispositions to CKD are evaluated, ensuring distinct, non-overlapping GWAS cohorts is crucial to prevent biased results. An example case study illustrating this principle is the recent MR analysis, which demonstrated the causal link between elevated lipoprotein(a) levels and CKD in European-ancestry populations [9].

In addition to selecting strong IVs, researchers must conduct sensitivity analyses to confirm the exclusion restriction assumption, which states that the IV should not affect the outcome except through the exposure. This is often tested using MR-Egger regression or MR-PRESSO to detect potential horizontal pleiotropy [35]. Additionally, screening PhenoScanner can effectively identify SNPs with potential pleiotropy [48]. For example, a high-quality MR study might report results from these tests, indicating minimal pleiotropy, thereby strengthening the credibility of causal inferences.

## 5. Sensitivity analyses: Ensuring robust causal inferences

Sensitivity analyses are vital components of MR studies, as they assess the robustness of findings and detect potential biases, particularly horizontal pleiotropy. Different methods, such as MR-Egger regression, MR-PRESSO, and the weighted median approach, each contribute unique strengths and limitations to the analysis, enabling researchers to cross-validate results and enhance causal inference reliability. Below, we explore these methods in detail.

### 5.1. MR-Egger regression: Advantages and limitations

MR-Egger regression is one of the most widely employed sensitivity analyses in MR studies [35]. It serves a dual purpose by testing for the presence of pleiotropy and providing an adjusted causal estimate. One of its key advantages is its ability to account for directional pleiotropy, where a genetic instrument may affect the outcome through pathways other than the exposure. This feature is especially useful when there is concern that multiple SNPs could introduce biases through non-exposure pathways [49].

Limitations:

- Reduced Statistical Power: MR-Egger regression often has less statistical power compared to other methods, making it less effective in studies with small sample sizes or when IVs only explain a small portion of the exposure’s variance.
- Potential Bias: The method may produce biased estimates if the instruments are weak, which could lead to misleading interpretations of causality.

### 5.2. MR-PRESSO: Detecting outliers and pleiotropy

MR-PRESSO (Mendelian Randomization Pleiotropy RESidual Sum and Outlier) is a powerful tool designed to detect and correct for horizontal pleiotropy by identifying outlier SNPs that disproportionately influence the results [50]. Once these outliers are removed, MR-PRESSO recalculates the causal estimate, offering a refined and potentially more accurate result.

Strengths:

- Pleiotropy Quantification: The ability of MR-PRESSO to quantify and correct for pleiotropy makes it highly effective for enhancing causal inference reliability.
- Transparency: Researchers should clearly report whether outliers were identified and how their removal impacted the final causal estimate.

Limitations:

- Instrument Requirements: MR-PRESSO requires a sufficient number of valid instruments to function effectively, limiting its application in studies with fewer available SNPs.
- Parameter Sensitivity: The results can be sensitive to the chosen parameters for outlier detection, potentially affecting robustness.

### 5.3. The weighted median approach

The weighted median approach is an additional sensitivity analysis that provides robust causal estimates even if up to 50% of the genetic instruments are invalid. This method acts as a complement to MR-Egger and MR-PRESSO by offering an alternative estimate that is less sensitive to outlier effects [35].

Advantages:

- Robustness: The weighted median method maintains accuracy even when some instruments do not meet all MR assumptions.
- Cross-Validation: By combining the weighted median approach with MR-Egger and MR-PRESSO, researchers can cross-validate their findings, mitigating biases inherent in any single method [35,51].

While individual sensitivity analyses offer distinct advantages, a comprehensive approach combining MR-Egger, MR-PRESSO, and weighted median methods provides the most robust evaluation of causal relationships. This multi-method strategy helps validate findings across different analytical frameworks. High-quality MR studies often report consistent findings across multiple sensitivity analyses to ensure robustness and reduce the risk of biases.

## 6. Challenges in conducting MR studies

Conducting robust MR studies comes with unique challenges that researchers and peer reviewers must consider to ensure methodological rigor and reliable conclusions.

### 6.1. Overlapping cohorts in GWAS data

One significant challenge in MR studies is the use of GWAS data from overlapping cohorts for both exposure and outcome groups. This overlap, common in large European datasets such as UK Biobank (UKB) and FinnGen, can lead to false-positive findings due to sample overlap, compromising the validity of causal inferences. Ensuring that the exposure and outcome cohorts are distinct is critical to avoid biased results [52]. Peer reviewers should scrutinize cohort information to confirm that overlapping populations have been effectively handled or avoided.

### 6.2. Racial and population differences

Certain kidney diseases, such as IgA nephropathy, exhibit significant racial and population differences, with notably higher prevalence in East Asian populations [53]. MR studies focusing on such diseases must prioritize data from populations where the disease prevalence aligns with the study’s focus. This improves the applicability and relevance of the findings and ensures that genetic instruments are population-specific, minimizing bias due to population stratification.

### 6.3. Selection of genetic IVs and addressing horizontal pleiotropy

The reliability of MR studies hinges on selecting robust genetic IVs and managing potential biases such as horizontal pleiotropy. As discussed in detail earlier, IVs must meet three key criteria: strong association with the exposure (*p* < 5 × 10^−8^) to accurately represent the exposure, exclusivity of causal pathway to adhere to the exclusion restriction assumption, and independence from confounding factors [54]. Researchers can verify this independence by consulting databases like the GWAS Catalog and PhenoScanner.

Horizontal pleiotropy remains a challenge even with thorough IV selection. To mitigate this, researchers should adopt a comprehensive analytical strategy that includes MR-Egger regression, MR-PRESSO, and the weighted median method, each offering distinct advantages for detecting and correcting pleiotropy [35].

### 6.4. Methodological and statistical considerations

Ensuring the robustness of MR analyses requires strict adherence to methodological standards:

- **F-statistics for Instrument Strength**: While many studies state that F-values exceed 10 to indicate strong instruments, specific F-statistics should be reported for each IV to enhance transparency [33,55].
- **Multivariable Mendelian Randomization (MVMR)**: When studying multiple exposures that may jointly influence an outcome, MVMR is necessary for accurate causal assessment [56,57].
- **Multiple Testing Corrections**: The use of corrections like the False Discovery Rate (FDR) or Benjamini-Hochberg method is critical for reducing false-positive findings, especially in studies evaluating multiple exposures or outcomes [58,59].
- **Statistical Power: **Based on significance level, effect size, sample size, and test type, the statistical power of an MR analysis can be calculated. This allows researchers to assess whether the sample size and effect size are sufficient to avoid false negatives, thereby enhancing the robustness of the conclusions [60].

### 6.5. Validation and replication of findings

Despite advancements, MR research still faces limitations in generalizability. Validation in independent cohorts and the use of negative control analyses are essential for confirming findings. Experimental validation should follow MR analyses to strengthen causal claims and uphold the scientific rigor expected in high-quality publications.

## 7. Critical appraisal: a checklist for peer reviewers

Appraising studies that use MR requires a thorough evaluation of the study’s methodology, validity, and interpretation of the results (Table 1). The first step is to assess the rationale and research question by ensuring the study poses a clear hypothesis with a rationale for using MR. In nephrology, this may involve evaluating causal relationships, such as whether elevated serum uric acid levels are causally linked to the risk of developing CKD or whether increased fibroblast growth factor 23 (FGF23) levels affect cardiovascular mortality in patients with end-stage kidney disease (ESKD). Next, examine the selection of genetic instruments (e.g. SNPs), ensuring they are strongly associated with the exposure, biologically plausible, and validated in GWAS. Instrument strength, commonly measured by F-statistics, should exceed 10 to avoid weak instrument bias.

**Table 1.** Structured approach to appraising Mendelian randomization studies (nephrology examples).

Category	Key Points	Nephrology Study Examples
**1. Rationale & Research Question**	• Clear hypothesis linking exposure and outcome.	• Example: Serum uric acid → CKD risk; FGF23 → Cardiovascular mortality in ESRD.
**2. Genetic Instrument Selection**	• SNPs should be strongly associated with exposure and biologically plausible.	• Example SNPs for serum uric acid or FGF23 levels.
**3. MR Assumptions**	• Relevance: SNPs must be associated with the exposure. • Independence: SNPs should not correlate with confounders. • Exclusion: SNPs affect outcome only *via* exposure.	• Use of SNPs associated with serum uric acid to assess risk of CKD or SNPs related to FGF23 for cardiovascular outcomes. • Independence of these SNPs from other CKD or ESRD confounders. • Testing for pleiotropy (MR-Egger, weighted median).
**4. Study Design & Methods**	• Use two-sample MR with consistent populations and sensitivity tests (MR-Egger, weighted median, MR-PRESSO).	• Apply two-sample MR to assess SNP-exposure (uric acid/FGF23) and outcome (CKD or cardiovascular outcomes). • Reverse causality test.
**5. Population & Data**	• Ensure adequate sample size; GWAS populations should reflect study cohort.	• GWAS data on CKD, ESRD, or cardiovascular diseases with sufficient sample sizes.
**6. Pleiotropy & Confounders**	• Minimize pleiotropy *via* MR-Egger; control confounding (e.g. PCA for stratification).	• Assess pleiotropy in studies of uric acid → CKD. • PCA for controlling population stratification.
**7. Statistical Analyses**	• Report causal estimates with confidence intervals; correct for multiple tests (e.g. Bonferroni).	• Causal effects of uric acid on CKD or FGF23 on cardiovascular outcomes. • Reporting confidence intervals and p-values in nephrology studies.
**8. Interpretation & Causal Inference**	• Confirm biological relevance; ensure findings align with known biology.	• Causality inferred from SNPs on nephrology markers (uric acid, FGF23) should match known pathophysiology.
**9. Limitations and Biases**	• Discuss limitations (e.g. pleiotropy, weak instruments) and generalizability.	• Address weak instruments or pleiotropy in CKD studies; generalize findings to broader CKD/ESRD populations.
**10. Replication and Validation**	• Validate findings in independent populations and with alternative instruments.	• Replication in separate CKD or ESRD cohorts; validate with alternative SNPs.
**11. Reviewer Action**	• Verify manuscript follows the STROBE-MR checklist for standard, transparent reporting.	• Ensure studies follow checklist for consistency in genetic instrument selection, pleiotropy tests, and replication.

**Abbreviations:** CKD (Chronic Kidney Disease); ESRD (End-Stage Renal Disease); FGF23 (Fibroblast Growth Factor 23); GWAS (Genome-Wide Association Studies); MR (Mendelian Randomization); MR-Egger (Mendelian Randomization-Egger); MR-PRESSO (Mendelian Randomization Pleiotropy RESidual Sum and Outlier); PCA (Principal Component Analysis); SNP (Single Nucleotide Polymorphism).

The assumptions of MR must also be evaluated. This includes confirming that the genetic instruments are relevant to the exposure, independent of confounders, and affect the outcome only through the exposure (not *via* pleiotropy). The study design, including the use of two-sample MR and sensitivity analyses like MR-Egger or MR-PRESSO, should be robust and consistent. Assess the population size and generalizability, ensuring the GWAS population reflects the target group. Additionally, consider pleiotropy and confounders, particularly through methods like principal component analysis (PCA). Review the statistical analyses, ensuring effect sizes, confidence intervals, and proper corrections for multiple testing are reported. Finally, interpretation of causality should align with biological plausibility, and limitations or biases must be clearly addressed. High-quality MR studies encourage replication and validation in different populations or contexts to strengthen the findings.

For peer reviewers assessing this type of study, a high-quality MR study will use well-validated, strong genetic instruments with clear biological relevance, ensuring the instruments are robustly associated with the exposure. It will thoroughly test and correct for pleiotropy and confounding factors, utilizing methods like MR-Egger to address potential biases. Sensitivity analyses, such as MR-PRESSO, should be applied to ensure the findings remain consistent across various assumptions. The study should clearly report effect sizes, accompanied by confidence intervals, while discussing the results within the context of established biological mechanisms. Additionally, limitations must be transparently addressed, along with recommendations for replication in future studies, to strengthen the findings. Applying this approach ensures the study’s design, analysis, and conclusions are sound, providing reliable causal inferences.

## 8. Biases and limitations in Mendelian randomization studies

MR studies are effective tools for inferring causal relationships but come with certain limitations and potential biases. Horizontal pleiotropy is a primary challenge, where genetic variants used as IVs may affect the outcome through alternative pathways unrelated to the exposure, leading to biased estimates. Addressing this requires employing robust sensitivity analyses such as MR-Egger regression and MR-PRESSO to detect and correct for pleiotropy. Additionally, weak instruments—IVs that are not strongly associated with the exposure—can compromise the validity of findings. Ensuring that each IV has an F-statistic greater than 10 helps maintain the strength of the instruments and reduce bias [61].

The validity of MR studies hinges on adherence to core assumptions: relevance, independence, and exclusion restriction. Violating these assumptions, particularly exclusion restriction, can lead to biased results due to confounding [62]. Therefore, researchers must select robust genetic instruments validated by large-scale GWAS and take steps to control for population stratification through methods such as PCA. Comprehensive sensitivity analyses, including approaches like the weighted median, MR-Egger, and MR-PRESSO, ensure that findings are cross-validated and biases minimized. Transparent reporting of effect sizes, confidence intervals, and multiple testing corrections enhances the credibility and reproducibility of MR results.

## 9. Emerging trends and future directions in MR research

As MR continues to evolve, researchers are increasingly adopting more sophisticated methodologies to address complex research questions and enhance the reliability of causal inferences. One significant advancement is MVMR, a method that allows for the simultaneous analysis of multiple exposures that may jointly influence an outcome. This approach is particularly useful in understanding the interplay between related risk factors, such as lipid levels, blood glucose, and blood pressure, in conditions like CKD [63]. By incorporating multiple variables into the model, MVMR can help isolate the independent effects of each exposure while adjusting for potential confounding among them, providing a clearer picture of causality.

Another promising development in MR research is the application of MR methodologies that incorporate machine learning and high-dimensional data [64]. These approaches are designed to identify relevant genetic instruments and potential pleiotropic pathways in large, complex datasets. Additionally, Bidirectional MR is gaining traction for its ability to test reverse causality by analyzing whether an outcome could causally affect the exposure. This is crucial in clarifying the directionality of relationships, particularly in conditions where causality may be ambiguous.

The field is also embracing more rigorous sensitivity analyses and pleiotropy-robust methods to mitigate biases and improve reliability. Techniques such as MR-Egger bootstrap and Radial MR are being used to better handle pleiotropy and improve the robustness of findings [65,66]. Moreover, there is a growing emphasis on applying multiple testing corrections, such as the FDR or Benjamini-Hochberg correction, to enhance the reliability of results in studies that evaluate multiple exposures or outcomes.

Future MR research will advance through multi-omics integration, specifically combining genetic data with proteomics and metabolomics to map detailed causal pathways. This integration will enable precise identification of molecular mechanisms underlying kidney disease progression and treatment response. Additionally, MR studies are expected to expand in global diversity by incorporating data from non-European populations, addressing current limitations related to population stratification and improving the generalizability of findings. These emerging trends and methodologies underscore the dynamic nature of MR research. Continued innovation and methodological refinement will ensure that MR remains a powerful tool for uncovering causal relationships and informing clinical practice in nephrology and beyond.

## 10. Conclusion

MR has revitalized nephrology research by providing a powerful approach to explore causal relationships between exposures and outcomes. However, it also presents challenges that researchers, journals, and peer reviewers must navigate carefully. The discernment of high-quality MR studies, underpinned by rigorous methodology and thorough sensitivity analyses, is essential for driving significant progress in nephrology. Conversely, an unchecked increase in low-quality MR studies risks diluting the scientific value of published work and may mislead future research and clinical applications. Maintaining methodological integrity and promoting robust MR research practices will uphold the credibility and impact of this tool in nephrology.

## Data Availability

All data that support this study has been provided and are also available on request from the corresponding author.

## References

[1] KDIGO 2024 clinical practice guideline for the evaluation and management of chronic kidney disease. Kidney Int. 2024;105(4):S117–s314. doi: 10.1016/j.kint.2023.10.018. 38490803

[2] Zhang R, Li Y, Nie Z. A bibliometric analysis from 2014 to 2024 reveals research hotspots and trends in the immunotherapy for glomerulonephritis. Hum Vaccin Immunother. 2024;20(1):2420446. doi: 10.1080/21645515.2024.2420446. 39494494 PMC11540077

[3] Nguyen K, Mitchell BD. A guide to understanding mendelian randomization studies. Arthritis Care Res (Hoboken). 2024;76(11):1451–1460. doi: 10.1002/acr.25400. 39030941 PMC11833605

[4] Krisanapan P, Pattharanitima P, Thongprayoon C, et al. Recent advances in understanding of cardiovascular diseases in patients with chronic kidney disease. J Clin Med. 2022;11(16):4653. doi: 10.3390/jcm11164653. 36012887 PMC9409994

[5] Liu C, Shen J, Ding Z, et al. Association between hypothyroidism and nephrotic syndrome: a bidirectional two-sample Mendelian randomization analysis. Ren Fail. 2024;46(2):2390558. doi: 10.1080/0886022X.2024.2390558. 39143823 PMC11328803

[6] Zhang Y, Zhong Z, Tang Z, et al. Insomnia and sleep duration for kidney function: Mendelian randomization study. Ren Fail. 2024;46(2):2387430. doi: 10.1080/0886022X.2024.2387430. 39132818 PMC11321106

[7] Song S, Sun Y, Yu J. Causal relationship between 731 immune cells and the risk of diabetic nephropathy: a two‑sample bidirectional Mendelian randomization study. Ren Fail. 2024;46(2):2387208. doi: 10.1080/0886022X.2024.2387208. 39091101 PMC11299454

[8] Lin R, Chen R. Exploring the causal connection: insights into diabetic nephropathy and gut microbiota from whole-genome sequencing databases. Ren Fail. 2024;46(2):2385065. doi: 10.1080/0886022X.2024.2385065. 39090986 PMC11299436

[9] Zhu Y, Chen S, Chen Z, et al. Causal effect of lipoprotein(a) level on chronic kidney disease of European ancestry: a two-sample Mendelian randomization study. Ren Fail. 2024;46(2):2383727. doi: 10.1080/0886022X.2024.2383727. 39082753 PMC11293262

[10] Shu J, Ge Y, Wu Y. Causal role of immune cells in IgA nephropathy: a mendelian randomization study. Ren Fail. 2024;46(2):2381593. doi: 10.1080/0886022X.2024.2381593. 39039855 PMC11268262

[11] Qing J, Zhang L, Li C, et al. Mendelian randomization analysis revealed that albuminuria is the key factor affecting socioeconomic status in CKD patients. Ren Fail. 2024;46(2):2367705. doi: 10.1080/0886022X.2024.2367705. 39010847 PMC11776065

[12] Zheng G, Cheng Y, Wang C, et al. Elucidating the causal nexus and immune mediation between frailty and chronic kidney disease: integrative multi-omics analysis. Ren Fail. 2024;46(2):2367028. doi: 10.1080/0886022X.2024.2367028. 39010723 PMC11265307

[13] Jing S, Lin L, Li J, et al. Causal relationship between Helicobacter pylori infection and IgA nephropathy: a bidirectional two-sample mendelian randomization study. Ren Fail. 2024;46(2):2371055. doi: 10.1080/0886022X.2024.2371055. 38946159 PMC467090

[14] Zhou B, Hong M, Jin L, et al. Exploring the relationship between creatine supplementation and renal function: insights from Mendelian randomization analysis. Ren Fail. 2024;46(2):2364762. doi: 10.1080/0886022X.2024.2364762. 38874125 PMC11232645

[15] Dong W, Li Q, Chen L, et al. Association between the gut microbiota and diabetic nephropathy: a two-sample Mendelian randomization study. Ren Fail. 2024;46(2):2357746. doi: 10.1080/0886022X.2024.2357746. 38832498 PMC11151794

[16] Chen Z, Zheng Z, Jiang B, et al. Genetic association between celiac disease and chronic kidney disease: a two-sample Mendelian randomization study. Ren Fail. 2024;46(2):2357246. doi: 10.1080/0886022X.2024.2357246. 38832490 PMC11151793

[17] Wu J, Zhang J, Huang G, et al. Evidence from mendelian randomization identifies several causal relationships between primary membranous nephropathy and gut microbiota. Ren Fail. 2024;46(1):2349136. doi: 10.1080/0886022X.2024.2349136. 38770992 PMC11110878

[18] Wen C, Chen L, Jia D, et al. Recent advances in the application of Mendelian randomization to chronic kidney disease. Ren Fail. 2024;46(1):2319712. doi: 10.1080/0886022X.2024.2319712. 38522953 PMC10913720

[19] Xiao R, Dong L, Xie B, et al. A Mendelian randomization study: physical activities and chronic kidney disease. Ren Fail. 2024;46(1):2295011. doi: 10.1080/0886022X.2023.2295011. 38178379 PMC10773648

[20] Zhou L-T, Ali AE, Jayachandran M, et al. Association between Kidney Stones and CKD: a bidirectional Mendelian randomization study. JASN. 2024;35(12):1746–1757. doi: 10.1681/ASN.0000000000000453. 39102294 PMC11617471

[21] Tang C, Chen P, Xu L-L, et al. Circulating proteins and IgA nephropathy: a multiancestry proteome-wide Mendelian randomization study. J Am Soc Nephrol. 2024;35(8):1045–1057. doi: 10.1681/ASN.0000000000000379. 38687828 PMC11377805

[22] Tin A, Köttgen A. Mendelian randomization analysis as a tool to gain insights into causes of diseases: a primer. J Am Soc Nephrol. 2021;32(10):2400–2407. doi: 10.1681/ASN.2020121760. 34135084 PMC8722812

[23] Khan A, Lim TY, Sanna-Cherchi S. Mendelian randomization unveils drug targets for IgA nephropathy. J Am Soc Nephrol. 2024;35(8):988–991. doi: 10.1681/ASN.0000000000000434. 38995687 PMC11377791

[24] Skrivankova VW, Richmond RC, Woolf BAR, et al. Strengthening the reporting of observational studies in epidemiology using Mendelian randomization: the STROBE-MR statement. JAMA. 2021;326(16):1614–1621. doi: 10.1001/jama.2021.18236. 34698778

[25] Burgess S, Davey Smith G, Davies NM, et al. Guidelines for performing Mendelian randomization investigations: update for summer 2023. Wellcome Open Res. 2019;4:186. doi: 10.12688/wellcomeopenres.15555.1. 32760811 PMC7384151

[26] Liu W, Zhang J, Zhang D, et al. Role of circulating inflammatory protein in the development of diabetic renal complications: proteome-wide Mendelian randomization and colocalization analyses. Front Endocrinol (Lausanne). 2024;15:1406442. doi: 10.3389/fendo.2024.1406442. 39040677 PMC11260607

[27] Ma Y, Ji J, Liu X, et al. Integrative analysis by Mendelian randomization and large-scale single-cell transcriptomics reveals causal links between B cell subtypes and diabetic kidney disease. Kidney Dis (Basel). 2024;10(5):327–345. doi: 10.1159/000539689. 39430286 PMC11488840

[28] Zhang C, Deng J, Li K, et al. Causal association of monocytes with chronic kidney disease and the mediation role of frailty: a study integrating large-scale two-sample Mendelian randomization and single-cell analysis. Arch Gerontol Geriatr. 2024;123:105435. doi: 10.1016/j.archger.2024.105435. 38583266

[29] Davies NM, Holmes MV, Davey Smith G. Reading Mendelian randomisation studies: a guide, glossary, and checklist for clinicians. BMJ. 2018;362:k601. doi: 10.1136/bmj.k601. 30002074 PMC6041728

[30] Lawlor DA, Harbord RM, Sterne JA, et al. Mendelian randomization: using genes as instruments for making causal inferences in epidemiology. Stat Med. 2008;27(8):1133–1163. doi: 10.1002/sim.3034. 17886233

[31] Allman PH, Aban I, Long DM, et al. A novel Mendelian randomization method with binary risk factor and outcome. Genet Epidemiol. 2021;45(5):549–560. doi: 10.1002/gepi.22387. 33998053

[32] Zhu Z, Zheng Z, Zhang F, et al. Causal associations between risk factors and common diseases inferred from GWAS summary data. Nat Commun. 2018;9(1):224. doi: 10.1038/s41467-017-02317-2. 29335400 PMC5768719

[33] Burgess S, Thompson SG, CRP CHD Genetics Collaboration. Avoiding bias from weak instruments in Mendelian randomization studies. Int J Epidemiol. 2011;40(3):755–764. doi: 10.1093/ije/dyr036. 21414999

[34] Burgess S, Thompson SG. Bias in causal estimates from Mendelian randomization studies with weak instruments. Stat Med. 2011;30(11):1312–1323. doi: 10.1002/sim.4197. 21432888

[35] Qiu-Qiang Z, Wei-Wei Y, Shan-Shu H, et al. Mendelian randomization of individual sleep traits associated with major depressive disorder. J Affect Disord. 2024;365:105–111. doi: 10.1016/j.jad.2024.08.068. 39153551

[36] Bowden J, Davey Smith G, Burgess S. Mendelian randomization with invalid instruments: effect estimation and bias detection through Egger regression. Int J Epidemiol. 2015;44(2):512–525. doi: 10.1093/ije/dyv080. 26050253 PMC4469799

[37] Slob EAW, Groenen PJF, Thurik AR, et al. A note on the use of Egger regression in Mendelian randomization studies. Int J Epidemiol. 2017;46(6):2094–2097. doi: 10.1093/ije/dyx191. 29025040

[38] Burgess S, Woolf B, Mason AM, et al. Addressing the credibility crisis in Mendelian randomization. BMC Med. 2024;22(1):374. doi: 10.1186/s12916-024-03607-5. 39256834 PMC11389083

[39] Burgess S, Butterworth A, Thompson SG. Mendelian randomization analysis with multiple genetic variants using summarized data. Genet Epidemiol. 2013;37(7):658–665. doi: 10.1002/gepi.21758. 24114802 PMC4377079

[40] Verbanck M, Chen CY, Neale B, et al. Detection of widespread horizontal pleiotropy in causal relationships inferred from Mendelian randomization between complex traits and diseases. Nat Genet. 2018;50(5):693–698. doi: 10.1038/s41588-018-0099-7. 29686387 PMC6083837

[41] VanderWeele TJ, Tchetgen EJ, Cornelis M, et al. Methodological challenges in mendelian randomization. Epidemiology. 2014;25(3):427–435. doi: 10.1097/EDE.0000000000000081. 24681576 PMC3981897

[42] Wu S, Kong M, Song Y, et al. Ethnic disparities in bidirectional causal effects between serum uric acid concentrations and kidney function: trans-ethnic Mendelian randomization study. Heliyon. 2023;9(11):e21108. doi: 10.1016/j.heliyon.2023.e21108. 37908715 PMC10613891

[43] Boef AG, Dekkers OM, Le Cessie S. Mendelian randomization studies: a review of the approaches used and the quality of reporting. Int J Epidemiol. 2015;44(2):496–511. doi: 10.1093/ije/dyv071. 25953784

[44] Palmer TM, Lawlor DA, Harbord RM, et al. Using multiple genetic variants as instrumental variables for modifiable risk factors. Stat Methods Med Res. 2012;21(3):223–242. doi: 10.1177/0962280210394459. 21216802 PMC3917707

[45] Tsao H-M, Lai T-S, Chang Y-C, et al. Serum urate and risk of chronic kidney disease: A Mendelian randomization study using Taiwan biobank. Mayo Clin Proc. 2023;98(4):513–521. doi: 10.1016/j.mayocp.2023.01.004. 36870858

[46] Burgess S, Small DS, Thompson SG. A review of instrumental variable estimators for Mendelian randomization. Stat Methods Med Res. 2017;26(5):2333–2355. doi: 10.1177/0962280215597579. 26282889 PMC5642006

[47] Dehghan A, Köttgen A, Yang Q, et al. Association of three genetic loci with uric acid concentration and risk of gout: a genome-wide association study. Lancet. 2008;372(9654):1953–1961. doi: 10.1016/S0140-6736(08)61343-4. 18834626 PMC2803340

[48] Kamat MA, Blackshaw JA, Young R, et al. PhenoScanner V2: an expanded tool for searching human genotype-phenotype associations. Bioinformatics. 2019;35(22):4851–4853. doi: 10.1093/bioinformatics/btz469. 31233103 PMC6853652

[49] Burgess S, Thompson SG. Interpreting findings from Mendelian randomization using the MR-Egger method. Eur J Epidemiol. 2017;32(5):377–389. doi: 10.1007/s10654-017-0255-x. 28527048 PMC5506233

[50] Ong JS, MacGregor S. Implementing MR-PRESSO and GCTA-GSMR for pleiotropy assessment in Mendelian randomization studies from a practitioner’s perspective. Genet Epidemiol. 2019;43(6):609–616. doi: 10.1002/gepi.22207. 31045282 PMC6767464

[51] Hemani G, Bowden J, Davey Smith G. Evaluating the potential role of pleiotropy in Mendelian randomization studies. Hum Mol Genet. 2018;27(R2):R195–r208. doi: 10.1093/hmg/ddy163. 29771313 PMC6061876

[52] Cho Y, Haycock PC, Sanderson E, et al. Exploiting horizontal pleiotropy to search for causal pathways within a Mendelian randomization framework. Nat Commun. 2020;11(1):1010. doi: 10.1038/s41467-020-14452-4. 32081875 PMC7035387

[53] Qu S, Zhou XJ, Zhang H. Genetics of IgA nephrology: risks, mechanisms, and therapeutic targets. Pediatr Nephrol. 2024;39(11):3157–3165. doi: 10.1007/s00467-024-06369-7. 38600219

[54] Chen LG, Tubbs JD, Liu Z, et al. Mendelian randomization: causal inference leveraging genetic data. Psychol Med. 2024;54(8):1461–1474. doi: 10.1017/S0033291724000321. 38639006

[55] Vansteelandt S, Lange C. Causation and causal inference for genetic effects. Hum Genet. 2012;131(10):1665–1676. doi: 10.1007/s00439-012-1208-9. 22864952

[56] Sanderson E, Davey Smith G, Windmeijer F, et al. An examination of multivariable Mendelian randomization in the single-sample and two-sample summary data settings. Int J Epidemiol. 2019;48(3):713–727. doi: 10.1093/ije/dyy262. 30535378 PMC6734942

[57] Burgess S, Thompson SG. Multivariable Mendelian randomization: the use of pleiotropic genetic variants to estimate causal effects. Am J Epidemiol. 2015;181(4):251–260. doi: 10.1093/aje/kwu283. 25632051 PMC4325677

[58] Wang Y, Hu X, Wang X, et al. Exploring the two-way link between migraines and venous thromboembolism: A bidirectional two-sample Mendelian randomization study. Thromb Haemost. 2024;124(11):1053–1060. doi: 10.1055/a-2313-0311. 38657649 PMC11518614

[59] Hemani G, Zheng J, Elsworth B, et al. The MR-Base platform supports systematic causal inference across the human phenome. Elife. 2018;7:e34408. doi: 10.7554/eLife.34408. PMC5976434 29846171

[60] Brion MJ, Shakhbazov K, Visscher PM. Calculating statistical power in Mendelian randomization studies. Int J Epidemiol. 2013;42(5):1497–1501. doi: 10.1093/ije/dyt179. 24159078 PMC3807619

[61] Davey Smith G, Hemani G. Mendelian randomization: genetic anchors for causal inference in epidemiological studies. Hum Mol Genet. 2014;23(R1):R89–98. doi: 10.1093/hmg/ddu328. 25064373 PMC4170722

[62] Gupta V, Walia GK, Sachdeva MP. Mendelian randomization’: an approach for exploring causal relations in epidemiology. Public Health. 2017;145:113–119. doi: 10.1016/j.puhe.2016.12.033. 28359378

[63] Lanktree MB, Thériault S, Walsh M, et al. HDL cholesterol, LDL cholesterol, and triglycerides as risk factors for CKD: A Mendelian randomization study. Am J Kidney Dis. 2018;71(2):166–172. doi: 10.1053/j.ajkd.2017.06.011. 28754456

[64] Ho YS, Fülöp T, Krisanapan P, et al. Artificial intelligence and machine learning trends in kidney care. Am J Med Sci. 2024;367(5):281–295. doi: 10.1016/j.amjms.2024.01.018. 38281623

[65] Slob EAW, Burgess S. A comparison of robust Mendelian randomization methods using summary data. Genet Epidemiol. 2020;44(4):313–329. doi: 10.1002/gepi.22295. 32249995 PMC7317850

[66] Bowden J, Spiller W, Del Greco M F, et al. Improving the visualization, interpretation and analysis of two-sample summary data Mendelian randomization via the Radial plot and Radial regression. Int J Epidemiol. 2018;47(6):2100–2100. doi: 10.1093/ije/dyy265. 30423109 PMC6280936

